# Identification and Classification of Parkinsonian and Essential Tremors for Diagnosis Using Machine Learning Algorithms

**DOI:** 10.3389/fnins.2022.701632

**Published:** 2022-03-21

**Authors:** Xupo Xing, Ningdi Luo, Shun Li, Liche Zhou, Chengli Song, Jun Liu

**Affiliations:** ^1^Shanghai Institute for Minimally Invasive Therapy, School of Health Science and Engineering, University of Shanghai for Science and Technology, Shanghai, China; ^2^Department of Neurology and Institute of Neurology, Ruijin Hospital, Shanghai Jiao Tong University School of Medicine, Shanghai, China

**Keywords:** Parkinsonian tremor, essential tremor, tremor differentiation, machine learning algorithms, upper limb posture

## Abstract

Due to overlapping tremor features, the medical diagnosis of Parkinson’s disease (PD) and essential tremor (ET) mainly relies on the clinical experience of doctors, which often leads to misdiagnosis. Seven predictive models using machine learning algorithms including random forest (RF), eXtreme Gradient Boosting (XGBoost), support vector machine (SVM), logistic regression (LR), ridge classification (Ridge), backpropagation neural network (BP), and convolutional neural network (CNN) were evaluated and compared aiming to better differentiate between PD and ET by using accessible demographics and tremor information of the upper limbs. The tremor information including tremor acceleration and surface electromyogram (sEMG) signals were collected from 398 patients (PD = 257, ET = 141) and then were used to train the established models to separate PD and ET. The performance of the models was evaluated by indices of accuracy and area under the curve (AUC), which indicated the ensemble learning models including RF and XGBoost showed the best overall predictive ability with accuracy above 0.84 and AUC above 0.90. Furthermore, the relative importance of sex, age, four postures, and five tremor features was analyzed and ranked showing that the dominant frequency of sEMG of flexors, the average amplitude of sEMG of flexors, resting posture, and winging posture had a greater impact on the diagnosis of PD, whereas sex and age were less important. These results provide a reference for the intelligent diagnosis of PD and show promise for use in wearable tremor suppression devices.

## Introduction

Parkinson’s disease (PD) and essential tremor (ET) are two common diseases usually accompanied by tremors of the upper limbs, which may severely impair motor function and have a negative influence on patients, especially in the aging population (Helmich et al., 2013). The symptoms of PD are complex and severe in the later stages; therefore, early diagnosis and effective treatment are crucial (Mark, 2007).

Owing to overlapping tremor features, it remains difficult to distinguish between PD and ET (Algarni and Fasano, 2018). Given that there is currently no gold standard to differentiate between PD and ET, the diagnosis of the two diseases mainly relies on the clinical experience of doctors (Thenganatt and Jankovic, 2016). Individuals diagnosed with PD typically have gradual development of non-motor symptoms for years before movement symptoms begin, but often they will not mention these symptoms unless specifically queried (Armstrong and Okun, 2020). Dopamine replacement therapy works better to diagnose PD. However, it could be difficult in the early stage of the disease and thus approximately a quarter of PD are misdiagnosed as ET, which usually causes the optimal medical treatments of the two diseases to be overlooked (Rizzo et al., 2016; Reich and Savitt, 2019; Armstrong and Okun, 2020).

Some efficient and accessible non-invasive biomarkers such as tremor signals including tremor acceleration and surface electromyogram (sEMG) have been investigated for the differentiation between PD and ET (Meigal et al., 2013; Barrantes et al., 2017). And a series of statistical characteristics of tremor signals including the dominant frequency and peak value were extracted and studied for distinguishing PD and ET (Hossen et al., 2010; Thanawattano et al., 2015; De Oliveira Andrade et al., 2020).

Artificial intelligence technology is widely used to solve problems in the medical field, including differentiating between PD and ET (Xiao et al., 2019; Duque et al., 2020). Based on various extracted statistical characteristics of tremor signals and methodologies of machine learning, a series of machine learning algorithms, such as linear models (logistic regression, ridge classification, etc.), ensemble learning models (random forest, XGBoost, etc.), the kernel-based model (support vector machine, etc.), and neural network models (backpropagation neural network, convolutional neural network, etc.) have been introduced for the diagnosis and progression prediction of PD and ET (Ai et al., 2011; Hossen, 2013; Ahmadi Rastegar et al., 2019; Hssayeni et al., 2019; Qin et al., 2019).

Tremors of the upper limbs in PD patients are mainly manifested as a resting tremor which can be used as an important symptom to distinguish between PD and ET, however, only 20% of ET patients suffer from that (Oren Cohen et al., 2003; Jankovic, 2008; Helmich et al., 2013). In addition to resting posture, stretching posture and some novel postures were introduced and investigated to evaluate their ability to discriminate PD from ET showing that tremors information collected from various postures behaves more effectively in differentiating between PD and ET compared to a single posture (Zhang et al., 2018).

Although research has been carried out by using tremor information of the upper limbs to differentiate PD and ET, the influence of various upper limb postures, tremor features, and demographics on the diagnosis has been rarely studied. To help clinicians better distinguish between PD and ET, we evaluated and compared seven prediction models using machine learning algorithms. Based on the results, we analyzed and compared the relative importance of various upper limb postures, tremor features, and demographics in the diagnosis of the two diseases.

## Materials and Methods

### Subjects and Data Collection

A total of 398 patients confirmed PD or ET with upper limb tremors were recruited for the experiment from June 2020 to November 2020 by the Department of Neurology of Rui Jin Hospital (Shanghai, China). With the help of a medical device system (Dantec^®^ Keypoint^®^ G4, Natus Medical Inc.), the tremor information, including acceleration and sEMG, was collected from four postures for each subject. And most of the subjects were tested on medication.

**FIGURE 1:**
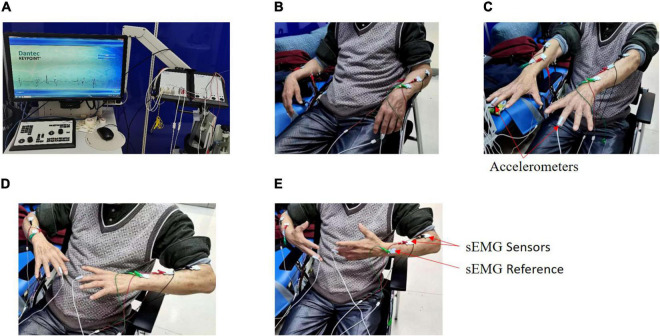
Experimental setup. Tremor information was collected from four postures by a medical device system called Dantec^®^ Keypoint^®^ G4 for each patient. **(A)** Dantec^®^ Keypoint^®^ G4. **(B)** Resting posture. **(C)** Stretching posture. **(D)** Winging posture. **(E)** Vertically winging posture.

Two accelerometers were fixed onto the distal finger of both hands, respectively, and six sEMG sensors were fixed onto the extensor and flexor muscles on both sides. In this experiment, each patient performed four respective postures (Figure 1): resting, stretching, winging, and vertically winging, meanwhile acceleration measurements and sEMG measurements were acquired.

For each patient, the sensor signals were measured for 30 s in each posture and sampled at a rate of 12,000 Hz. Patients were asked to avoid unrelated behaviors, and irrelevant personnel were removed from the room throughout the experiment. The demographics of age and sex for each patient were also recorded.

For each posture, five tremor features (each tremor feature with two tremor variables), including the dominant frequency of the acceleration signals, the dominant frequency of sEMG (extensor), the dominant frequency of sEMG (flexor), the average amplitude of sEMG (extensor), and the average amplitude of sEMG (flexor), were acquired by the Dantec^®^ Keypoint^®^ G4 medical device system. Finally, a total of 40 tremor variables (Table 1) were obtained from the four postures. Our study was approved by the local ethics committee of Shanghai Jiao Tong University.

**TABLE 1:** Demographic data of 398 patients.

Cases (*n* = 398, Male 196, Female 22)			Mean	SD
Age			66.23	40.85
Resting posture	Dominant frequency	Acc (L)	3.41	3.17
		Flexor (L)	8.83	4.21
		Extensor (L)	8.77	4.26
		Acc (R)	3.48	2.97
		Flexor (R)	8.31	4.53
		Extensor (R)	8.95	4.11
	Average amplitude	Flexor (L)	212.38	173.45
		Extensor (L)	201.94	119.85
		Flexor (R)	202.28	120.14
		Extensor (R)	171.96	88.05
Stretching posture	Dominant frequency	Acc (L)	3.99	2.90
		Flexor (L)	9.21	4.30
		Extensor (L)	10.33	4.55
		Acc (R)	3.38	2.78
		Flexor (R)	9.38	3.93
		Extensor (R)	10.23	4.53
	Average amplitude	Flexor (L)	167.54	76.38
		Extensor (L)	203.56	73.17
		Flexor (R)	173.75	97.12
		Extensor (R)	210.59	76.67
Winging posture	Dominant frequency	Acc (L)	6.25	2.33
		Flexor (L)	7.59	4.09
		Extensor (L)	9.18	4.43
		Acc (R)	3.53	2.53
		Flexor (R)	8.68	3.91
		Extensor (R)	10.35	5.56
	Average amplitude	Flexor (L)	196.71	103.60
		Extensor (L)	213.69	79.56
		Flexor (R)	188.90	119.53
		Extensor (R)	217.25	81.16
Vertically winging posture	Dominant frequency	Acc (L)	4.17	2.33
		Flexor (L)	8.77	3.95
		Extensor (L)	9.70	4.33
		Acc (R)	3.66	7.88
		Flexor (R)	8.55	3.96
		Extensor (R)	9.68	4.39
	Average amplitude	Flexor (L)	196.01	98.92
		Extensor (L)	190.21	84.42
		Flexor (R)	182.90	83.32
		Extensor (R)	188.41	70.05

*SD, standard deviation; L, left; R, right.*

*Acc (L) affiliated to “Dominant frequency” attached to “Resting posture”: the dominant frequency of the acceleration signal collected from the left hand; Flexor (L) affiliated to “Dominant frequency” attached to “Resting posture”: the dominant frequency of the surface EMG signal collected from the flexor on the left hand; Extensor (L) affiliated to “Dominant frequency” attached to “Resting posture”: the dominant frequency of the surface EMG signal collected from the extensor on the left hand. And the others have similar meanings.*

### Establishment of Models

Based on several predictive models widely adopted in many clinical applications, seven predictive models, including random forest (RF), eXtreme gradient boosting (XGBoost), support vector machine (SVM), backpropagation neural network (BP), ridge classification (Ridge), logistic regression (LR), and convolution neural network (CNN), were established and compared to differentiate PD and ET using tremor information collected from upper limbs.

For the linear models, LR and Ridge were selected. For the ensemble learning models, such as RF and XGBoost, multiple evaluators were established using the sample, and an output response was obtained after considering and aggregating the results of multiple evaluators. And a traditional machine learning algorithm, SVM, was built. Finally, the neural network models, including BP and CNN, were selected due to their powerful non-linear learning ability and extensive application to diagnose and predict the progression of PD (Hossen, 2013).

Because of different principles and usage between the CNN model and the other six models, the raw sensor signals, including the acceleration measurement and sEMG measurement of upper limbs, were used to train the CNN model to differentiate PD and ET. Due to the large volume of the time-series data which needs to be further processed for CNN, we did not combine demographic data to train the model. For the other six models, 40 tremor variables acquired from the Dantec^®^ Keypoint^®^ G4 medical device system, as well as two demographics (sex and age), were used to train these models. Therefore, for CNN and the other six models, the data preprocessing and training of the models were different.

For these six models (RF, XGBoost, SVM, BP, Ridge, and LR), data preprocessing was performed as follows. For each patient, 40 tremor variables and two demographics (sex and age) were used as the variables with the diagnosis of either PD or ET as the labels, resulting in a total of 398 samples. Table 1 indicates the two demographics (sex and age) and a total of 40 tremor variables affiliated to four postures, with each posture having ten tremor variables. First, we filled in the null values with the mean value of each variable (Zheng and Casari, 2018; Géron, 2019). Then, we scaled the data using Z-score normalization (Eq. 1) to enhance the predictive ability of the model and prevent overfitting (Géron, 2019).


(1) $$z=\frac{x-u}{\sigma }$$

Where *u* is the mean of the variable and σ is the standard deviation.

**FIGURE 2:**
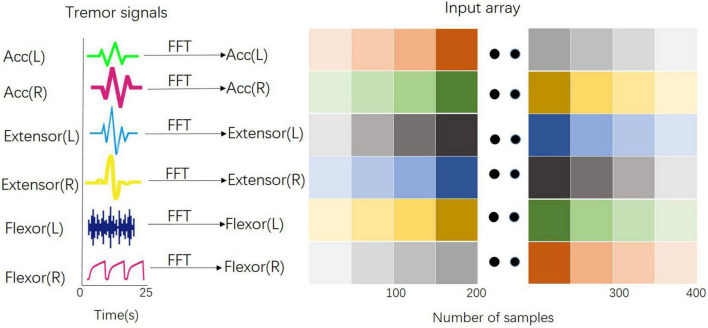
Input array for the training of the CNN model. All signals from the acceleration measurement and sEMG measurement have been converted into the frequency domain by the Fast Fourier Transform and stacked along the vertical axis to form a two-dimensional array for CNN input.

For the CNN model, data preprocessing was performed as follows. Raw acceleration and sEMG measurements were used to train the CNN model. The middle 25 s of each signal was selected to avert potential noise in the experimental procedure, and then the extracted data were down-sampled to 120 Hz for ease of calculation, following which these down-sampled signals were converted to the frequency domain using a fast Fourier transform (FFT). Because the frequency band of pathological tremors is mainly in the 2–20 Hz range, the FFT signals at 2–20 Hz were finally chosen. The 24 converted signals from the acceleration measurement and sEMG measurement were stacked along the vertical axis to form a two-dimensional array for CNN input (Figure 2), and they were scaled using Eq. 1 (Kim et al., 2018).

### Training of Models

Some parameters were selected and adjusted using the grid search method to acquire the best parameter combination for each model. Table 2 lists the technical parameters of the models. First, the data were preprocessed as described above and then randomly divided into a training set (80%) and a validation set (20%). The proportion of PD and ET in the training set was consistent with that in the validation set.

**TABLE 2:** Tuning parameters of the seven models.

Models	Tuning
RF	n_estimators (subtrees)
XGBoost	max_depth(maximum depth of number)
SVM	γ(Gaussian kernel), C(Cost)
BP	Size (hidden layer units); α(Regulation parameter)
Ridge	α(Regulation parameter)
LR	C (reciprocal of Regulation parameter)
CNN	The number of convolutional layers, the number of kernels

*RF, random forest; XGBoost, eXtreme Gradient Boosting; SVM, support vector machine; BP, backpropagation neural network; LR, logistic regression; Ridge, ridge classification; CNN, convolutional neural network.*

**FIGURE 3:**
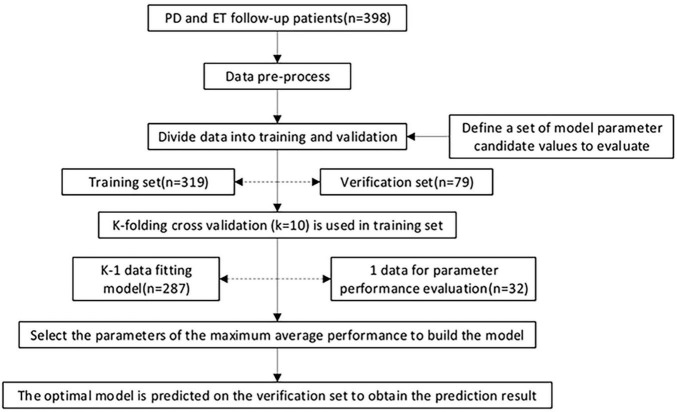
Model training, parameter adjustment, and performance evaluation. 398 patients were recruited in the current study. The data were pre-processed and randomly divided into a training set (80%) and a validation set (20%), and the proportion of the two class proportions in each set is the same. In the training set, k-fold cross-validation (*k* = 10) is used, and various parameter combinations are exhausted by grid search. Performance evaluation index of AUC was adopted to judge the average predictive performance of the model. The average performance maximum is used as the best performance tuning parameter, and the prediction is finally performed on the test set.

Ten-fold cross-validation was applied to the training set to obtain the optimal model parameters. The training set was divided into ten parts, nine of which were used to train the model in turn; the remaining one was used to test the model. The average value of AU-ROC, which was calculated ten times, was used as an indicator to evaluate the model for determining the different parameter combinations for each model. A forecast flow chart is shown in Figure 3. Because of the high sampling frequency and lack of good connectivity between muscles and sensors in some aged patients, some acceleration measurements or sEMG measurements were corrupted and became distorted, which led to only 188 samples could finally being used to train the CNN model.

**FIGURE 4:**
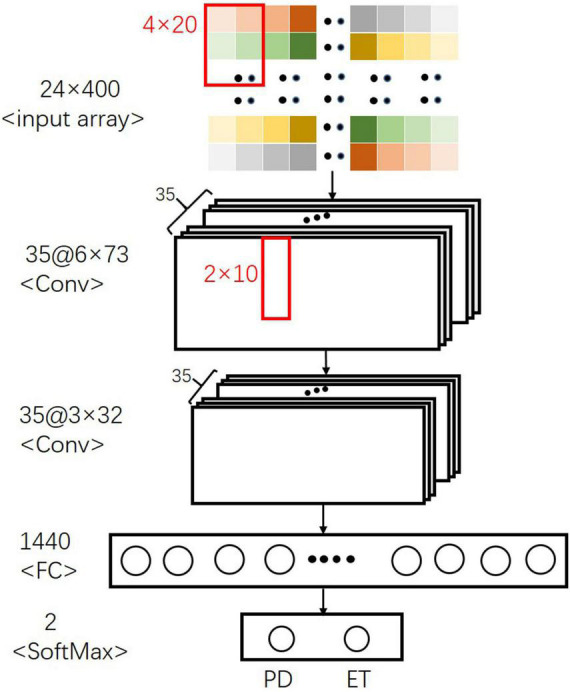
Final CNN architecture for separating PD from ET.

For the CNN model, a specially formulated structure (Figure 4) containing several layers of neural networks was established to distinguish between PD and ET. The first layer of the convolutional neural network received a normalized two-dimensional input array, and 4 × 20 convolution kernels with 4 × 5 strides were used to fuse the local signal information from a signal sensor with the output size of 6 × 73. The second convolutional layer with 2 × 10 convolution filters and 2 × 2 strides was used to extract the sensor information. After each convolutional layer, a batch normalization layer and a dropout layer with a 30% dropout rate were used to avoid overfitting. Finally, a fully connected layer and a softmax classifier were used to distinguish between PD and ET.

### Evaluation of Models

Evaluation indicators, including the confusion matrix, accuracy, area under the curve (AUC), recall (TPR, sensitivity), specificity, F1, false positive rate (FPR,1- specificity), and precision calculated by true positives (TP), false positives (FP), true negatives (TN), and false negatives (FN), were used to evaluate the performance of each model (Eqs 2–7). And higher AUC value indicates a better overall performance of the current feature, ς.


(2) $$T\,P\,R=\frac{T\,P}{T\,P+F\,N}$$

(3) $$F\,P\,R=\frac{F\,P}{F\,P+T\,N}$$

(4) $$P\,r\,e\,c\,i\,s\,i\,o\,n=\frac{T\,P}{T\,P+F\,P}$$

(5) $$A\,c\,c\,u\,r\,a\,c\,y=\frac{T\,P+T\,N}{T\,P+F\,P+F\,N+T\,N}$$

(6) $$A\,U\,C=\int_{-\infty }^{\infty }T\,P\,R\,\left(\varsigma \right)-F\,P\,R\,\left(\varsigma \right)\,d\,\varsigma$$

(7) $$F\,1=2\times \frac{R\,e\,c\,a\,l\,l\times P\,r\,e\,c\,i\,s\,i\,o\,n}{R\,e\,c\,a\,l\,l+P\,r\,e\,c\,i\,s\,i\,o\,n}$$

where AUC denotes the area under the curve value of the variable ς.

Furthermore, we analyzed the relative importance of the variables in each model, except for CNN. The models XGBoost and RF allowed the importance of variables to be derived during model training; the coefficients of the Ridge model were used as the importance factor.

For models, such as LR, BP, and SVM, wherein the importance of variables was difficult or impossible to extract, the mean decrease accuracy was obtained by directly measuring the effect of each feature on the accuracy of the model. Briefly, the model was fitted, and parameter adjustment was performed to predict the validation set to obtain the model performances. Then, the feature values were disturbed to establish a new disturbance prediction set. Obviously, for the unimportant variables, the scrambling order has little effect on the accuracy of the model, but for the important variables, the scrambled order will reduce the accuracy of the model. Finally, the relative importance ratio of all the eigenvalues was given a weight between 0 and 1 according to the overall proportion.

We added the relative importance of the ten tremor variables affiliated to each posture as the relative importance of the four postures, respectively. In addition, we added the relative importance of the two tremor variables affiliated to each tremor feature attached to the four postures as the relative importance of the five tremor features, respectively, thereby obtaining the effect sizes.

## Results

### Tuning of Parameters

**FIGURE 5:**
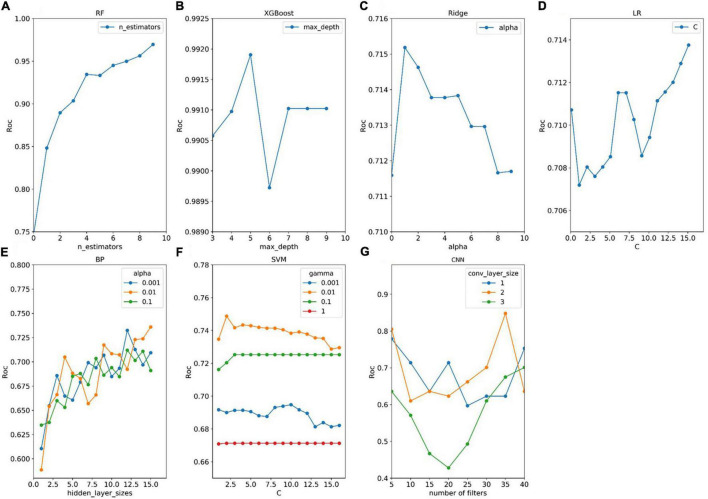
Tuning results of model parameters. **(A–G)** Four models (RF, XGBoost, Ridge, and LR) have one adjustment parameter, and three models (BP, SVM, and CNN) have two adjustment parameters. For each set of parameters, the model parameters were evaluated for fit using the procedure described in panel Figure 2. The optimal parameters for each model are selected by obtaining the parameters that the model evaluates to the maximum.

The average AU-ROC for different models and their parameters are listed (Figure 5). In these models, XGBoost obtained the best overall performance, and the parameter max_depth of five was optimal. RF achieved optimal performance as the parameter n_estimators reached nine. A two-layered CNN architecture with 35 convolution kernels was developed (Figure 4). The other four models had a similar performance, with a maximum performance index of approximately 0.7. The cost (C) of SVM was two, and the parameter gamma of 0.01 produced the best performance. For LR, parameter C (reciprocal of the regulation parameter) of 15 performed the best. For BP, parameter hidden layer sizes of 15 and an alpha of 0.01 produced the best performance. The alpha of the Ridge was one, which enabled the optimal performance.

### Validation of the Training Set

The confusion matrices of the seven models are displayed in Table 3. The number of actual subjects of PD and ET in the confusion matrix is 51 and 28, respectively.

**TABLE 3:** Confusion matrices of seven models.

Confusion matrix	Actual	Prediction	
		PD	ET
RF	PD	44	7
	ET	6	22
XGBoost	PD	49	2
	ET	10	18
SVM	PD	50	1
	ET	27	1
BP	PD	41	10
	ET	12	16
Ridge	PD	38	13
	ET	16	12
LR	PD	40	11
	ET	9	19
CNN	PD	19	3
	ET	5	10

*AUC, area under the curve; PD, Parkinson’s disease; ET, essential tremor.*

For RF and XGBoost, the sum of false negatives (FNs) and false positives (FPs) could be controlled within 13, while the others had a sum of FNs and FPs above 20 (79 validation samples). For CNN, the sum of FNs and FPs was eight (37 validation samples). The evaluation indices, including recall (TPR, sensitivity), specificity, accuracy, FPR (1-specificity), and F1 for each model, are displayed in Table 4. For the ensemble learning models, RF and XGBoost show a better performance, with an accuracy rate equal to and above 0.84. XGBoost has a higher accuracy rate than RF. However, the specificity of RF is higher, which means that it has a higher accuracy rate in identifying ET patients. For the neural networks, the accuracy of BP and CNN reaches 0.72 and 0.78, respectively. Compared with BP, the CNN model has a stronger non-linear predictive ability. In this study, the accuracy of CNN was also higher than that of BP. However, the neural network did not perform well owing to the limited number of samples. The Ridge linear model obtained the lowest accuracy rate of 0.63 and the lowest AU-ROC value of 0.71.

**TABLE 4:** Evaluation summary based on AUC, recall, specificity, accuracy, FPR and precision.

Models	AUC	Recall	Specificity	Accuracy	FPR	Precision	F1
RF	0.90	0.86	0.79	0.84	0.21	0.88	0.87
XGBoost	0.95	0.96	0.64	0.85	0.36	0.83	0.89
SVM	0.81	0.98	0.04	0.65	0.96	0.65	0.78
BP	0.75	0.80	0.57	0.72	0.43	0.77	0.78
Ridge	0.71	0.75	0.43	0.63	0.57	0.70	0.72
LR	0.73	0.78	0.68	0.75	0.32	0.82	0.80
CNN	0.77	0.86	0.67	0.78	0.33	0.79	0.83

*FPR, false positive rate.*

### Important Features

**FIGURE 6:**
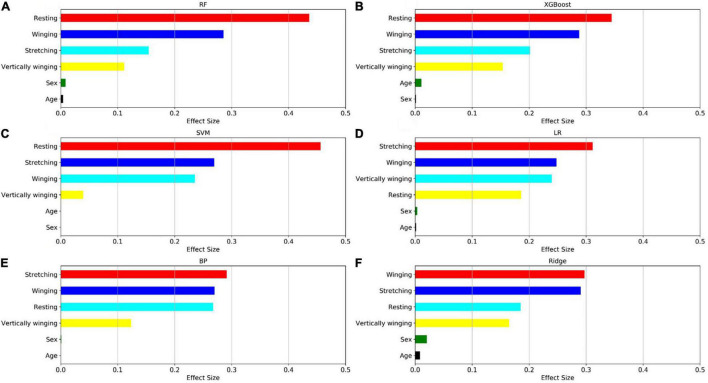
Factors effect size. The **(A–F)** histogram displays the proportion of the factoric importance of sex, age, and four postures calculated by the models. For each model, the relative importance is quantified by assigning a weight between 0 and 1 for each variable and then the relative importance of the four postures is calculated by the sum of the factoric importance of the corresponding variables affiliated to that posture. The models XGBoost and RF allow the importance of variables to be derived during model training; the coefficients of the Ridge model are used as the basis for factor importance; the LR, BP, and SVM models are obtained by the Mean decrease accuracy method.

**FIGURE 7:**
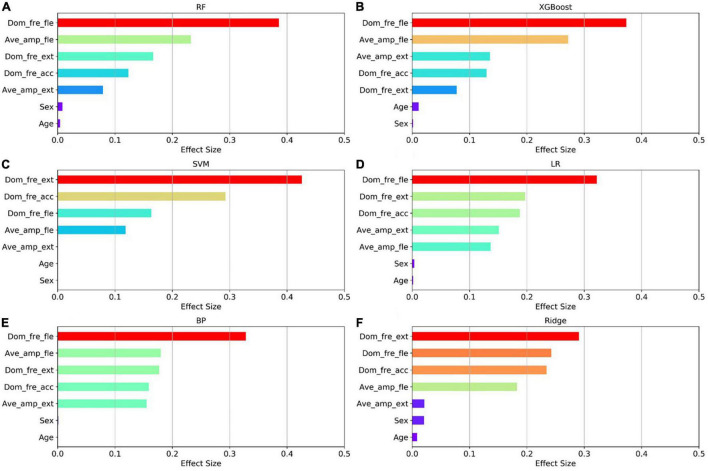
Factors effect size. The **(A–F)** histogram displays the proportion of the factoric importance of sex, age, and five tremor features calculated by the models. For each model, the relative importance is quantified by assigning a weight between 0 and 1 for each variable and then the relative importance of the five tremor features is calculated by the sum of the factoric importance of the corresponding variables affiliated to that tremor feature. The models XGBoost and RF allow the importance of variables to be derived during model training; the coefficients of the Ridge model are used as the basis for factor importance; the LR, BP, and SVM models are obtained by the Mean decrease accuracy method.

The relative importance of sex, age, and the four postures (resting, stretching, winging, and vertically winging), were calculated using the models displayed in Figure 6. The relative importance of sex, age, and the five tremor features, including the dominant frequency of acceleration of distal fingers (Dom_fre_acc), the dominant frequency of sEMG of extensors (Dom_fre_ext), the dominant frequency of sEMG of flexors (Dom_fre_fle), the average amplitude of sEMG of extensors (Ave_amp_ext), and the average amplitude of sEMG of flexors (Ave_amp_fle), were calculated by the models as displayed in Figure 7.

Among the seven established models, the ensemble learning models, including RF and XGBoost showed the best prediction capabilities. Thus, the relative importance obtained from these two models was adopted. In the two models, the relative levels of importance of sex, age, the four postures, and the five tremor features were ranked showing that resting posture, winging posture, Dom_fre_fle, and Ave_amp_fle had a significant influence on the predictability of the models, whereas sex and age had a slight impact on the prediction.

## Discussion

Most PD and ET patients suffer from tremors of the upper limbs (Zhang et al., 2018; Duque et al., 2020). Owing to the overlapping tremor features, misdiagnosis between PD and ET is common. As a non-invasive biomarker, the tremor information of upper limbs, including acceleration and sEMG, has been investigated to distinguish PD from ET. Although some tremor features (tremor amplitude, dominant frequency, etc.) from various upper limb postures are extracted for the differentiation of PD and ET, the relative importance of the tremor features and various upper limb postures have been less frequently investigated.

In this study, we applied the tremor signals, including the acceleration measurements and sEMG measurements, which were collected from the four upper limb postures and two demographics (sex and age) to distinguish PD from ET using seven machine learning algorithms. The ensemble learning models RF and XGBoost provided a rapid classification of outpatients. Various complex models could be established, and accurate decisions could be made using machine learning algorithms when given certain data. In this study, we used a dataset with a size of 398 and 42 dimensions. It was proved that the ensemble learning models performed better than the other models and fulfilled the clinical needs.

It may be considered that the current sample was not sufficient to support the result owing to the limited sample size. In the case of small data size and high data dimensions, the ensemble learning classifier XGBoost and RF could separate samples more effectively, whereas the other models of SVM, LR, BP, Ridge, and CNN exhibited a lower accuracy. Owing to the high data dimensions, SVM had a low predictive ability, resulting in most samples being predicted as PD, and Ridge had the lowest accuracy rate. The more complex neural network model with a powerful non-linear learning ability also did not perform well. In this study, among the seven established models, the ensemble learning models RF and XGBoost performed ideally, while the other five models lacked a significant predictive ability.

Although some assistive engineering approaches using tremor information of the upper limbers collected by wearable sensors have been proposed to differentiate between PD and ET, the results are less convincing limited by a few subjects. In this paper, we evaluated seven classification models using machine learning algorithms to differentiate PD and ET by using accessible demographics and tremor information of the upper limbs collected from various postures. The results with AUC above 0.90 and accuracy above 0.84 for RF and XGBoost models are convincing because more subjects (398 cases) were collected and the data was adequate compared with previous studies. Furthermore, we firstly analyzed and ranked the relative importance of sex, age, the four postures, and the five tremor features for differentiating PD and ET, which could help the diagnosis of PD in the early stage.

Recent progress in artificial intelligence and wearable technology has made wearable tremor suppression devices for PD a potentially viable alternative for tremor management. The relative importance of sex, age, the four postures, and the five tremor features, provides a reference for the intelligent diagnosis of PD and shows promise for use in wearable tremor suppression devices. To further enhance the performance of the established models, more ET subjects will be recruited in the subsequent study.

## Conclusion

In this study, seven models were evaluated and compared for separation of PD from ET by using the tremor information of the upper limbs in various postures. It was determined that the ensemble learning models, including RF and XGBoost, had the greatest overall predictive ability and could effectively distinguish PD and ET. We also found that the dominant frequency of flexor sEMG, the average amplitude of flexor sEMG, the resting posture, and the winging posture had a greater impact on the predictability of the models, whereas the other predictors, specifically sex and age, were less important. These results provide a reference for the intelligent diagnosis of PD and are promising for use in wearable tremor suppression devices. This study investigating the differentiation between PD and ET using machine learning algorithms was preliminary. With the further acquisition of data of ET subjects in future work, the performance of models will be further improved and more valuable results will be obtained.

## Data Availability Statement

The raw data supporting the conclusions of this article will be made available by the authors, without undue reservation.

## Ethics Statement

The studies involving human participants were reviewed and approved by the Local Ethics Committee of Shanghai Jiao Tong University. The patients/participants provided their written informed consent to participate in this study.

## Author Contributions

XX, NL, LZ, CS, and JL designed the experiments. XX and NL analyzed the dataset and drafted the manuscript. SL performed the experiments. All authors contributed to the article and approved the submitted version.

## Conflict of Interest

The authors declare that the research was conducted in the absence of any commercial or financial relationships that could be construed as a potential conflict of interest.

## Publisher’s Note

All claims expressed in this article are solely those of the authors and do not necessarily represent those of their affiliated organizations, or those of the publisher, the editors and the reviewers. Any product that may be evaluated in this article, or claim that may be made by its manufacturer, is not guaranteed or endorsed by the publisher.
